# Process optimization for green synthesis of silver nanoparticles by *Sclerotinia sclerotiorum* MTCC 8785 and evaluation of its antibacterial properties

**DOI:** 10.1186/s40064-016-2558-x

**Published:** 2016-06-24

**Authors:** Juhi Saxena, Prashant Kumar Sharma, Madan Mohan Sharma, Abhijeet Singh

**Affiliations:** Department of Biosciences, Manipal University Jaipur, Dehmi Kalan, Near GVK Toll Plaza, Jaipur-Ajmer Express Highway, Jaipur, Rajasthan 303007 India; Dr. B. Lal Institute of Biotechnology, Malviya Industrial Area Jaipur, Jaipur, India; Dr. M.P.S. College, Agra, India

**Keywords:** Green synthesis, *Sclerotinia sclerotiorum*, Silver nanoparticles, Optimization, Antibacterial activity

## Abstract

**Background:**

Eco-friendly synthesis of nanoparticles is viewed as an alternative to the chemical method and initiated the use of microorganisms for synthesis. The present study has been designed to utilize plant pathogenic fungi *Sclerotinia sclerotiorum* MTCC 8785 strain for synthesis and optimization of silver nanoparticles (AgNPs) production as well as evaluation of antibacterial properties. The AgNPs were synthesized by reduction of aqueous silver nitrate (AgNO_3_) solution after incubation of 3–5 days at room temperature. The AgNPs were further characterized using UV–visible spectroscopy, Fourier transform infrared spectroscopy (FTIR) and transmission electron microscopy (TEM). Reaction parameters including media, fungal biomass, AgNO_3_ concentration, pH and temperature were further optimized for rapid AgNPs production. The antibacterial efficacy of AgNPs was evaluated against *Escherichia coli* ATCC 25922 and *Staphylococcus aureus* ATCC 25923 by disc diffusion and growth kinetics assay at the concentration determined by the minimum inhibitory concentration (MIC).

**Results:**

AgNPs synthesis was initially marked by the change in colour from pale white to brown and was confirmed by UV–Vis spectroscopy. Optimization studies showed that potato dextrose broth (PDB) media, 10 g of biomass, addition of 2 mM AgNO_3_, pH 11 and 80 °C temperature resulted in enhanced AgNPs synthesis through extracellular route. TEM data revealed spherical shape AgNPs with size in the range of 10 nm. Presence of proteins capped to AgNPs was confirmed by FTIR. AgNPs showed antibacterial activity against *E. coli* and *S. aureus* at 100 ppm concentration, corresponding MIC value.

**Conclusion:**

*S. sclerotiorum* MTCC 8785 mediated AgNPs was synthesized rapidly under optimized conditions, which showed antibacterial activity.

**Electronic supplementary material:**

The online version of this article (doi:10.1186/s40064-016-2558-x) contains supplementary material, which is available to authorized users.

## Background

Nanotechnology, an emerging field of nanoscience deals with the synthesis and applications of nanoscale materials in diverse interdisciplinary fields like physics, chemistry, biology, medicine and agriculture (Albrecht et al. 2006). Silver is preferred over other metals in nanoparticle synthesis due to its strong antimicrobial action (Markowska et al. 2013).

Researchers are immensely interested in nanoparticles synthesis by physical or chemical means and nanoparticles synthesized through these routes are designated as engineered nanoparticles (ENPs). Moreover, improper disposal of ENPs lead to their exposure to environment and different ecosystems. In the past few years, interest in extracellular synthesis of nanoparticles mainly by fungi has been increased due to easy synthesis, less toxicity, less downstream processing and better optimization control (Pooja et al. 2014). In extracellular synthesis of silver nanoparticles from fungi, firstly biomass is allowed to grow in suitable medium. Fungi respond to different cultural conditions and compositions differently and secrete different metabolites and different kinds of proteins. Fully grown biomass is harvested and separated completely from media components, which is then transferred to deionized water. In water, enzymes or proteins and metabolites have been secreted by fungal biomass through reverse osmosis. In the subsequent stages, removal of biomass from deionized water (cell free filtrate) consists of specific enzymes which catalyze the reduction of aqueous silver ions for synthesis of AgNPs (Birla et al. 2013).

Several fungal species including *Fusarium oxysporum* (Karbasian et al. 2008), *Fusarium semitectum* (Basavaraja et al. 2008) *Saccharomyces boulardii* (Kaler et al. 2013), *Alternaria alternata* (Gajbhiye et al. 2009), *Aspergillus flavus* (Jain et al. 2011), *Penicillium brevicompactum* (Shaligram et al. 2009), *Xanthomonas oryzae* (Narayanan 2013) have been explored to synthesize AgNPs. Effect of culture medium on the extracellular synthesis of AgNPs using *Klebsiella pneumoniae*, *E. coli* and *Pseudomonas jessinii* have been studied recently (Muller et al. 2016). Authors have concluded that the formation of AgNPs results from the interaction of all medium components. In another study conducted by Morsy Fatthy (2015) have shown that dead bacteria are able to synthesize AgNPs by releasing organics. Both these studies concluded that microorganisms do not per se synthesize AgNPs, hence not biogenic.

Moreover, plant pathogenic fungi which are not harmful to humans can be exploited for nanoparticle synthesis through green chemistry for biomedical applications. In order to increase the yield and the shelf-life (stability) of AgNPs with minimum investment, it is necessary to optimize the cultural conditions and various physical parameters like pH and temperature.

Bacterial strains have been increasing at an alarming rate and represent a major threat to modern medicine. Emergence of antibiotic resistance is the consequence of a complex interaction of factors involved in the evolution and spread of resistance mechanisms (Holmes et al. 2016). Overuse and inappropriate usage of antibiotics led to the development of antibiotic resistance in clinics. In the recent times, nanomedicine has been evolved with immense potential due to its antimicrobial arsenal to combat pathogenic microbes. AgNPs, a potent bactericidal have been extensively used as bactericidal against Gram positive and Gram negative bacteria.

Presence of protein caps in nanoparticles help in stabilization and binding to cell surface receptor results in increased binding and uptake of drug or genetic material on human cells (Zhang et al. 2013). Hence it is worthwhile to explore the presence of capped protein in AgNPs synthesized from cell free filtrate of *S. sclerotiorum* MTCC 8785, a phytopathogen causing white mold diseases in plants.

The present study deals with the extracellular synthesis of AgNPs using plant pathogenic fungi using *S. sclerotiorum* MTCC 8785 followed by its characterization and optimization for rapid AgNPs synthesis. Furthermore, antibacterial studies have also been carried out. The study also explores the presence of capping material around the AgNPs.

## Methods

### Fungal strain and growth conditions

The fungal strain *S*. *sclerotiorum* MTCC 8785 was obtained from Microbial Type Culture Collection (MTCC), Chandigarh, India. *S*. *sclerotiorum* MTCC 8785 was grown on potato dextrose agar (PDA) at 28 °C for 96 h. The fungal strain was routinely maintained on PDA slants. The test organisms including Gram negative *E. coli* ATCC 25922 and Gram positive *S. aureus* ATCC 25923 were procured from Dr. B. Lal clinical laboratory private limited, Jaipur, India.

### Extracellular synthesis of AgNPs

The fungal mycelium grown on PDA was inoculated in production media [potato dextrose broth (PDB)] followed by incubation at 28 °C for 5 days. Fully grown mycelia were washed with sterile distilled water to remove media components. 5 g of washed mycelia were added to 10 ml of deionized water and agitated at 28 °C for 48 h. From the water, mycelia was removed by filtration and the filtrate was collected which is termed as cell free filtrate (CFF). CFF was incubated with 1 mM silver nitrate (AgNO_3_) followed by agitation in a shaker at 120 rpm at 30 °C in the dark for 3-5 days. A control set without AgNO_3_ was simultaneously agitated with experimental set (Chowdhury et al. 2014).

### Optimization studies for rapid AgNPs synthesis

Different reaction parameters like media, AgNO_3_ concentration, fungal biomass, pH and temperature were optimized to obtain maximum production of AgNPs. Inoculum containing 10^5^ spores/ml was inoculated in different media like Potato dextrose broth (PDB), Sabouraud’s dextrose (SB), Protease production media (PP), Czapek Dox (CZAPEK), Richard medium (RM) and Glucose Yeast Extract Peptone (GYP) was subjected to 1 mM AgNO_3_ and incubated at 30 °C. Different concentration range of AgNO_3_ (0.2–2 mM) was added to CFF obtained from fungal biomass grown in optimized media followed by incubation at 30 °C. Furthermore, fungi grown in optimized media with different biomass ranging from 0.5 to 10 gm was subjected to optimized concentration of AgNO_3_ and incubated at 30 °C to monitor AgNPs synthesis. After this, the optimum concentration of AgNO_3_ was added to the CFF at different pH values (3, 5, 7, 9 and 11) and incubated at 30 °C. For temperature optimization, CFF containing optimum AgNO_3_ concentration at optimum pH was incubated at 4–80 °C. The sample was analyzed with UV–visible absorption spectroscopy to confirm the synthesis of AgNPs (Nayak et al. 2011).

### Characterization of silver nanoparticles

#### UV–visible spectroscopy analysis

AgNO_3_ treated CFF of *S*. *sclerotiorum* MTCC 8785 were monitored for reduction of silver ions on UV–visible spectrophotometer 119 (Systronics).

#### FTIR spectroscopy analysis

To investigate the presence of capped protein around synthesized AgNPs, Fourier transform infrared (FTIR) spectroscopy was performed. CFF and AgNO_3_ treated CFF were freeze–dried and FTIR spectrum was recorded on FTIR (Shimazdu, India) in the range of 400–4000 cm^−1^ at a resolution of 4 cm^−1^.

#### TEM analysis

Morphological characterization of AgNPs was done to assess its shape and size. A drop of solution containing AgNPs was loaded on copper grids in transmission electron microscope (Jeol, USA) and analysis was done for size and shape.

### Antibacterial tests

The antimicrobial activity of AgNPs was evaluated using the minimum inhibitory concentration (MIC) method by broth dilution as per the guidelines of National Committee for Clinical Laboratory Standards (NCCLS). Firstly 1000 ppm of AgNPs solution was prepared by dissolving 1 mg of AgNPs in 1 ml sterile distilled water. Further it was diluted so as to get different concentrations. Test bacterial suspensions (*E. coli* and *S. aureus*) were diluted in sterile Muller Hinton broth to obtain final inoculums of 10^6^ CFU/ml. The culture flasks containing Muller Hinton broth were treated with various concentrations (6.25, 12.5 25, 50, 100, 200 and 400 ppm) of AgNPs followed by inoculation of *E. coli* and *S. aureus* (10^6^ CFU/ml). The samples were then incubated at 37 °C at 150 rpm for 24 h. The lowest concentration of AgNPs where no turbidity (visible growth of a microorganism) was observed as MIC. Culture medium containing AgNPs and without test organisms was used as negative control.

Furthermore, growth kinetics of test bacteria in presence of AgNPs was studied as described earlier (Ruparelia et al. 2008). Briefly, 10 ml of nutrient broth was treated with various concentrations (25–125 ppm) of AgNPs, inoculated with overnight grown test bacterial culture (10^6^ CFU/ml) and incubated for 24 h at 37 °C. Bacterial growth was estimated in spectrophotometer at 600 nm after 24 h of incubation. Absorbance of the mixture was recorded at 600-nm wavelength at regular time intervals (0, 4, 6, 8, 10, 12, and 24 h). Sample without AgNPs was used as negative control in this experiment. Ampicillin (10 ppm) and amikacin (30 ppm) were used as positive control against *E. coli* and *S. aureus* respectively.

The antibacterial property of AgNPs synthesized from *S*. *sclerotiorum* MTCC 8785 was further evaluated by disc diffusion method against test bacterial strains procured from Dr. B. Lal Clinical laboratory Pvt. Ltd, Jaipur. The bacterial inoculum was prepared by diluting the overnight culture with Mueller–Hinton (MH) broth and inoculated on MH agar according to Mc. Farland assay in accordance with CLSI guidelines. Paper discs soaked with different concentrations of AgNPs (25, 50, 75 and 100 ppm) were placed onto MH agar plates after swabbing with prepared inoculums of *E. coli* and *S. aureus* cultures. The plates were kept overnight at 37 °C and zone of inhibition was measured (Sarkar et al. 2007). CFF was used as negative control. Ampicillin (10 µg/disc) and amikacin (30 µg/disc) were used as positive control against *E. coli* and *S. aureus* respectively.

## Results and discussions

### Extracellular synthesis of AgNPs

**Fig. 1:**
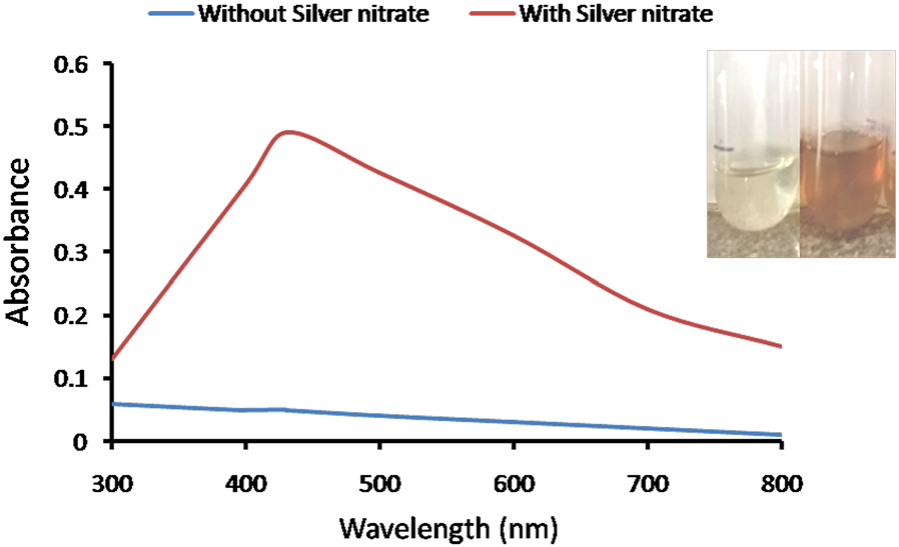
UV–Vis spectra of AgNPs synthesized using CFF of *S*. *sclerotiorum* MTCC 8785. *Inset* Change in *color* in CFF after addition of 1 mM AgNO_3_ at pH 7 followed by incubation at 30 °C

Synthesis of AgNPs from AgNO_3_ solution using CFF of *S*. *sclerotiorum* MTCC 8785 was observed by change in color of the solution (Fig. 1 inset). Color of the solution turned from colorless **(**Fig. 1 inset left panel**)** to brown **(**Fig. 1 inset right panel**)**, which indicated the formation of AgNPs. The appearance of brown color in the solution is indicates the reduction of silver ions to silver. This bioreduction is may be catalyzed by reducing agent secreted by the microorganism in the solution (Ahmad et al. 2003). Furthermore, the extracellular synthesis of AgNPs was confirmed in UV–visible spectroscopy. Absorption spectra showed a strong peak at 430 nm **(**Fig. 1**)**, indicated a surface plasmon resonance (SPR), having nanoparticles with sizes less than 100 nm. This could be due to excitation of electrons in the conductive band around the nanoparticles surface as reported by Busi et al. (2014).

### Optimization studies for AgNPs production

Optimization studies were done to support better growth of fungi as well as to enhance better yield of AgNPs. The growth conditions, such as media, AgNO_3_ concentration, biomass, pH, and temperature directly affecting the productivity were optimized.

#### Effect of media

**Fig. 2:**
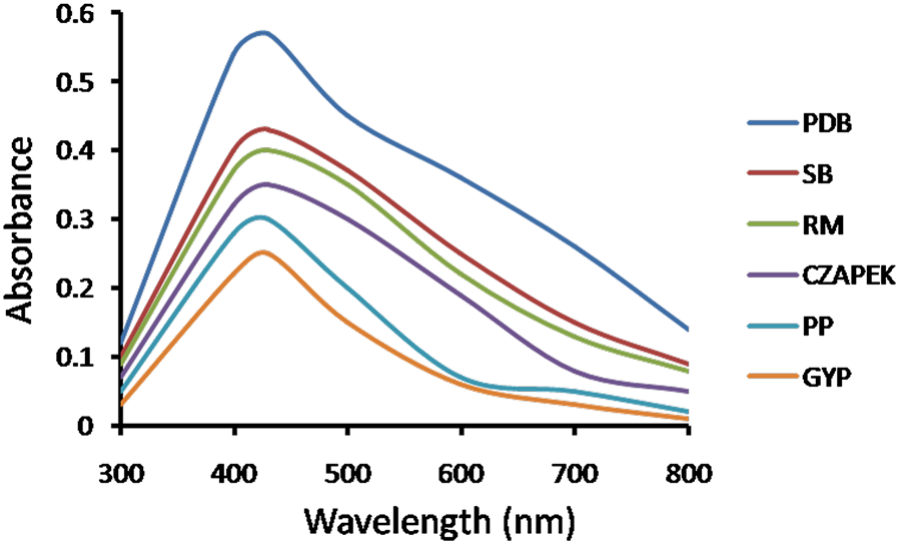
UV–Vis spectra of AgNPs synthesis using fungi grown in different media followed by addition of 1 mM AgNO_3_ in CFF of 5 gm biomass and incubated in pH 7 at 30 °C

A range of culture media have been used to grow fungal mycelia for enhanced extracellular synthesis of AgNPs. UV–Vis spectra showed a sharp peak at 430 nm. Fungal biomass grown in PDB has shown enhanced AgNPs synthesis followed by SB, RM, CZAPEK, PP and GYP **(**Fig. 2**)**. This may be due to presence of ingredients in PDB stimulating better growth of fungi and help in producing augmented level of reducing agent responsible for silver ion reduction (Birla et al. 2013).

#### Effect of AgNO_3_ concentration

**Fig. 3:**
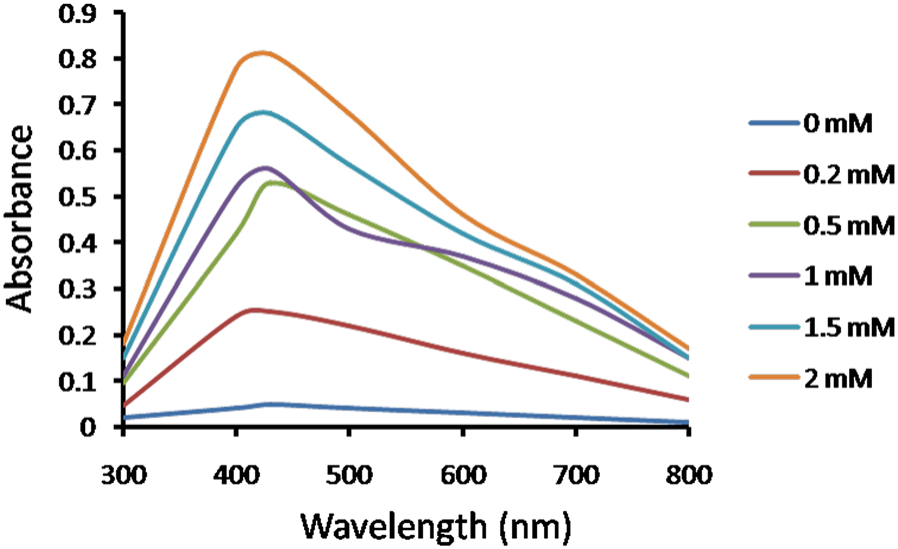
UV–Vis spectra of AgNPs synthesis using fungi grown in PDB media followed by addition of different concentration of AgNO_3_ in CFF of 5 gm biomass and incubated in pH 7 at 30 °C

In order to study the effect of substrate on nanoparticle production, different concentration of AgNO_3_ solution (0.2–2 mM) was used to synthesize AgNPs using *S*. *sclerotiorum* MTCC 8785. The synthesized AgNPs was monitored by UV–Vis spectral analysis and provided evidence that increase in concentration of AgNO_3_ up to 2 mM led to complete reduction of Ag^+^**(**Fig. 3**)**. Our results correlate with Dattu et al., which shows maximum AgNPs synthesis at 2 mM AgNO_3_ concentration using *Penicillium* sp (Singh et al. 2014).

#### Effect of biomass

**Fig. 4:**
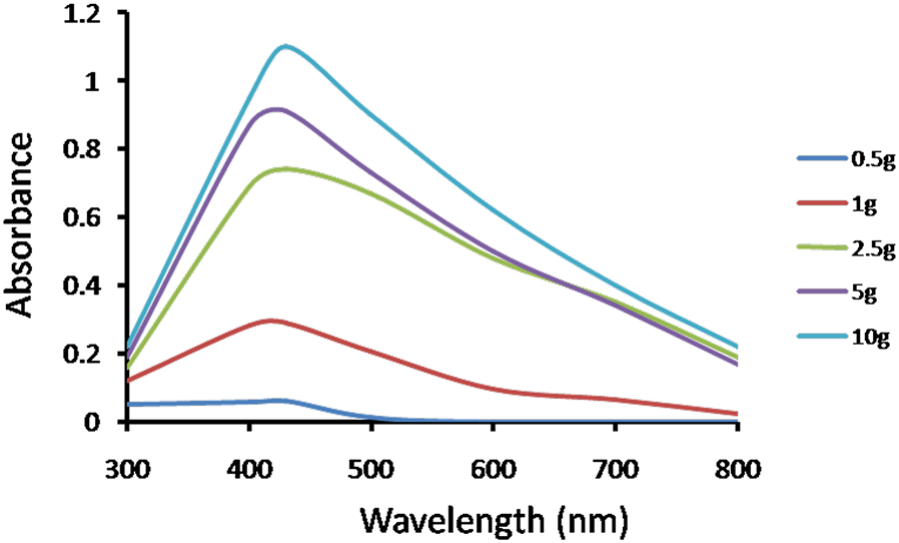
UV–Vis spectra of AgNPs synthesis using fungi grown in PDB media followed by addition of 2 mM AgNO_3_ in CFF from different fungal biomass and incubated in pH 7 at 30 °C

Extracellular synthesis of AgNPs was observed using different amount of fungal biomass ranging from 0.5 to 10 gm. UV–visible absorption spectra revealed as the amount of wet biomass increases, synthesis of AgNPs increases which is represented by peak at 430 nm **(**Fig. 4**)**. This can be due to directly proportional relationship between amount of biomass and release of reducing agent responsible for AgNPs synthesis (Birla et al. 2013).

#### Effect of pH

**Fig. 5:**
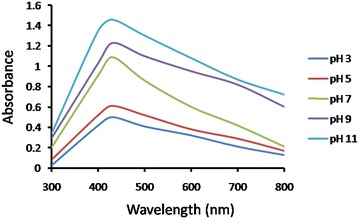
UV–Vis spectra of AgNPs synthesis using fungi (10 gm biomass) grown in PDB media followed by incubation in different pH at 30 °C

pH is an essential factor affecting AgNPs production. The effect of varying pH for maximum AgNPs synthesis, AgNO_3_ was added in the CFF having different pH (3, 5, 7, 9 and 11). The maximum production of AgNPs was attained at pH11 by change in color when compared with other pH values as demonstrated by UV–visible absorption spectra **(**Fig. 5**)**. Similar observations have been reported in case of *Pseudomonas aeruginosa* (Taheri et al. 2011) and *Trichoderma viride* (Chitra and Annadurai 2013) mediated AgNPs synthesis where high pH favor the complete reduction of Ag^+^ into AgNPs by providing electrons.

#### Effect of temperature

**Fig. 6:**
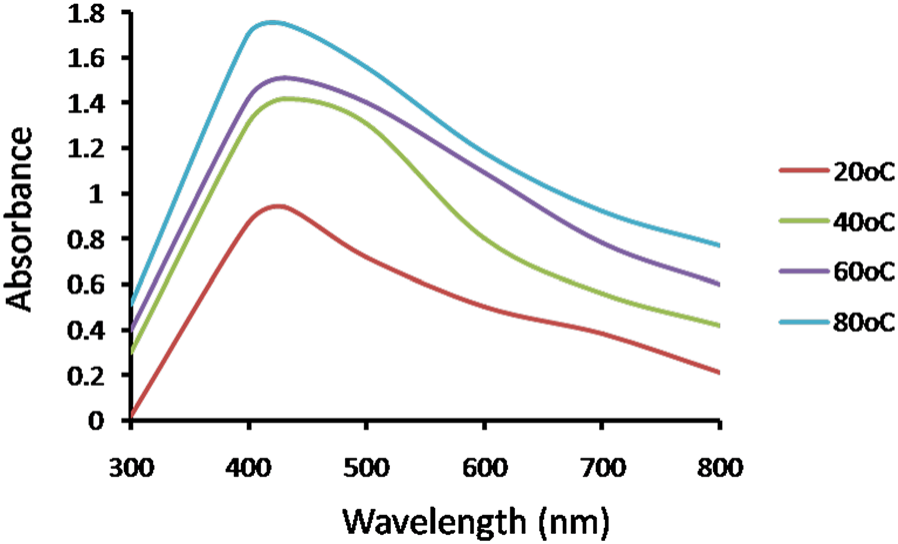
UV–Vis spectra of AgNPs synthesis using fungi grown in PDB media followed by addition of 2 mM AgNO_3_ in CFF of 10 gm biomass and incubated in pH 11 at different temperature

To evaluate the effect of temperature on AgNPs production by *S*. *sclerotiorum* MTCC 8785 CFF (pH 11) containing 2 mM AgNO_3_ was incubated at different temperatures from 20 to 80 °C with a difference of 20 °C and monitored for AgNPs synthesis. Maximum synthesis of AgNPs was observed at 80 °C and remains stable for longer time period indicated stabilized synthesis **(**Fig. 6**)**. High temperature imparts increased kinetic energy and lead to faster synthesis rate (Birla et al. 2013). After optimization, synthesis was observed within 12 h.

#### TEM characterization of synthesized AgNPs

**Fig. 7:**
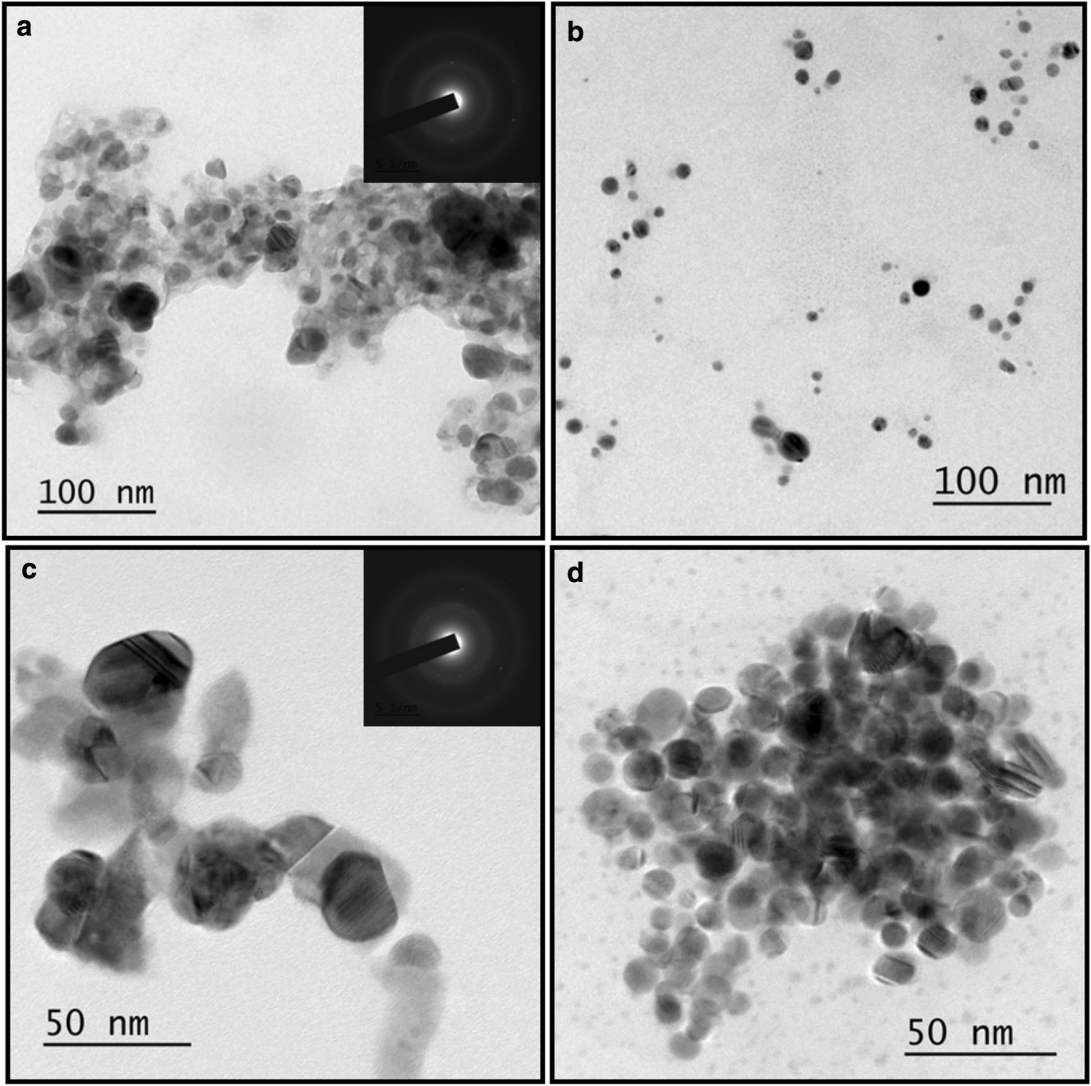
TEM images of AgNPs synthesized at 30 °C, pH 7 with 1 mM AgNO_3_ at low (**a**) and high magnification (**c**). TEM images for AgNPs synthesized at 80 °C, pH 11 with 2 mM AgNO_3_ at low (**b**) and high magnification (**d**)

The shape, size and morphology of AgNPs under optimized conditions was studied using TEM. TEM images reported the spherical shape of AgNPs with average particle size ranges from 25 to 30 nm **(**Fig. 7a, c**)**. However, when *S*. *sclerotiorum* MTCC 8785 was employed under optimized conditions (PDB media, 2 mM AgNO_3_, 10 gm biomass, pH 11 and temperature 80 °C), nanoparticles with average size in range of 10-15 nm were obtained (Fig. 7b, d). Selected area electron diffraction pattern (SAED) revealed the crystalline nature of AgNPs synthesised using *S*. *sclerotiorum* MTCC 8785 **(**Fig. 7a inset and c inset). In another study, conducted by Banu et al. (2011) revealed spherical shaped AgNPs, with the size ranging between 3 and 20 nm by *Rhizopus stolonifer*.

#### FTIR characterization of synthesized AgNPs

**Fig. 8:**
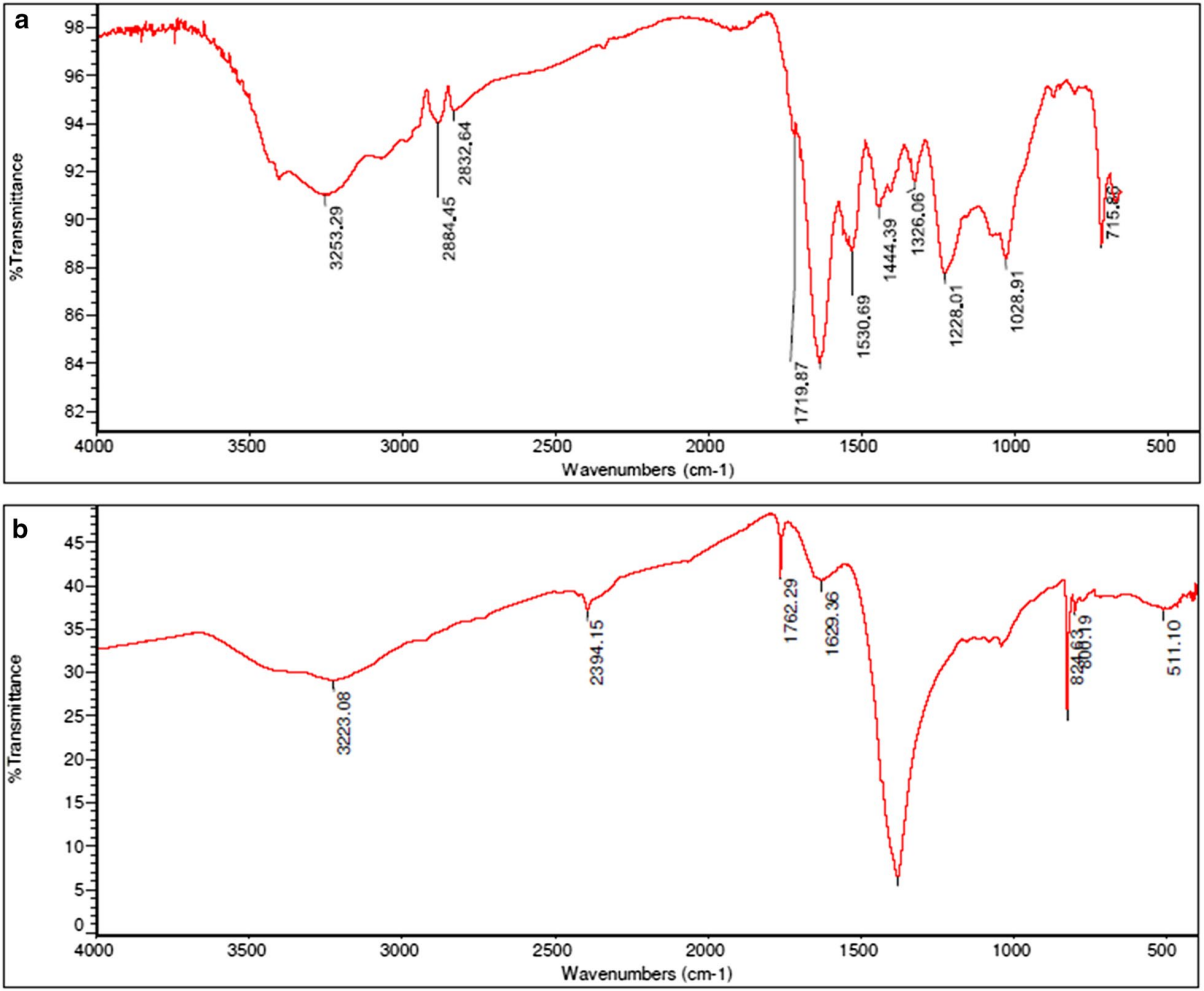
FTIR spectra of **a** cell free filtrate and **b** synthesized AgNPs

FTIR measurement of CFF and synthesized AgNPs was carried out to identify the functional groups present on AgNPs and proteins surrounding AgNPs as stabilization agent. The absorption peaks of cell free filtrate was located at 715, 1028, 1228, 1326, 1444, 1530, 1637, 1719, 2832, 2884 and 3253 cm^−1^ (Fig. 8a). However FTIR spectrum of mixture containing CFF and AgNO_3_ shows peak shifts with intense absorption bands at 511, 800, 824, 1379, 1629, 1762, 2394 and 3223 cm^−1^ (Fig. 8b**)**. Presence of peak at 3223 and 3253 cm^−1^ may be assigned to N–H stretch which corresponds to primary and secondary amines. Presence of band at 23,884 and 2390 cm^−1^ correspond to –C≡N stretching vibrations. The peaks seen at 1762, 1719 and 1629 cm^−1^ assigned to C=O and N–H bend (primary amine) of peptide linkage respectively. Difference in peaks in CFF and mixture containing CFF and AgNO_3_ suggested that reaction between biomolecules present in CFF with AgNO_3_ has occurred and new product in the form of AgNPs have been formed. FTIR study indicates that probably the carboxyl (–C=O) and amine (N–H) groups in CFF are mainly involved in the reduction of Ag^+^ ions to AgNPs. Furthermore, FTIR analysis also confirmed the presence of proteins around the AgNPs acting as reducing as well as stabilizing agent during synthesis of AgNPs (Daima et al. 2013; Venkatesan et al. 2014) (Additional file 1: Fig. S1).

### Antibacterial efficacy

**Fig. 9:**
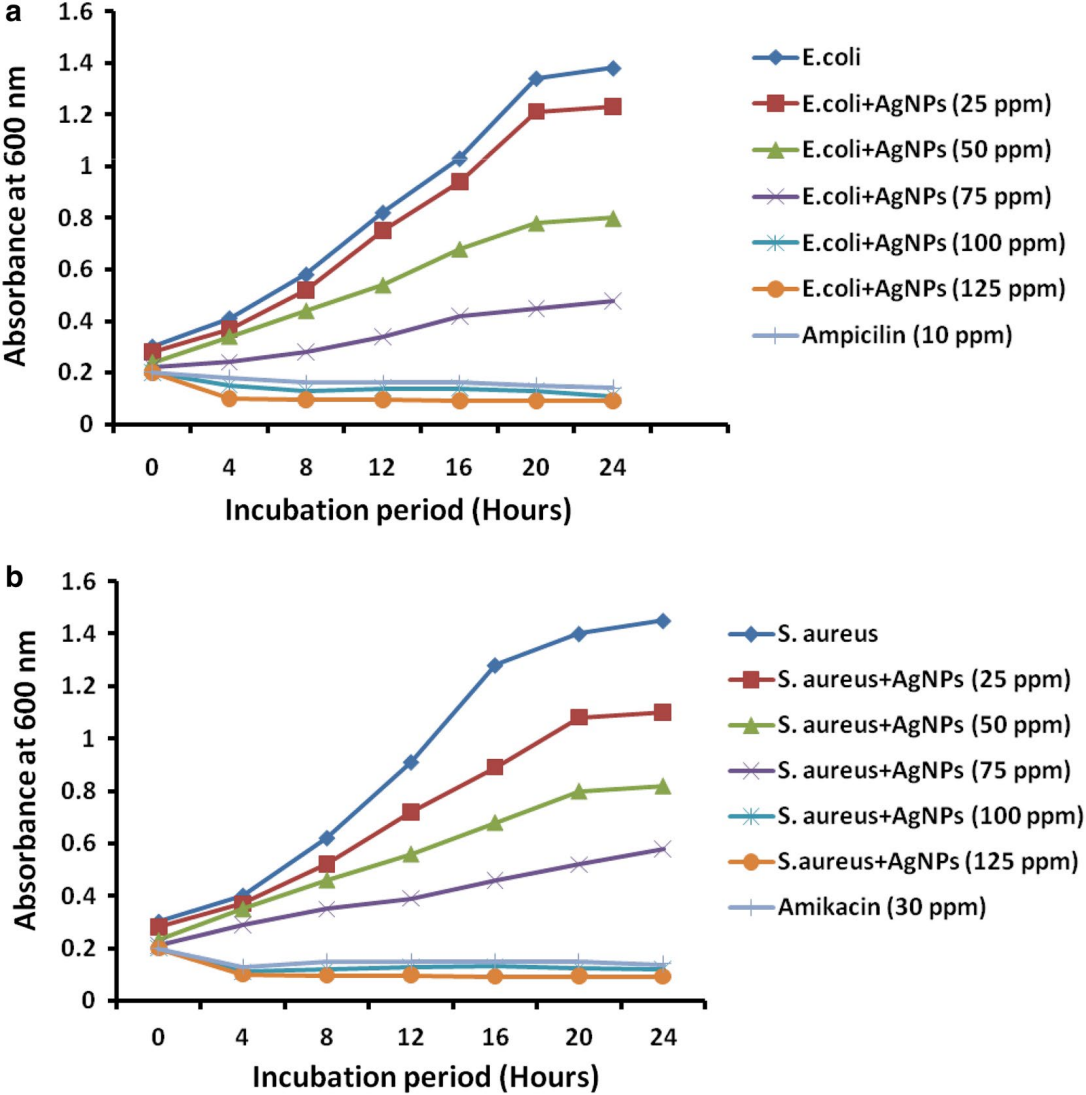
Growth profiles of **a** *E. coli* and **b** *S. aureus* in the presence of varying amounts of AgNPs (25–125 ppm)

**Fig. 10:**
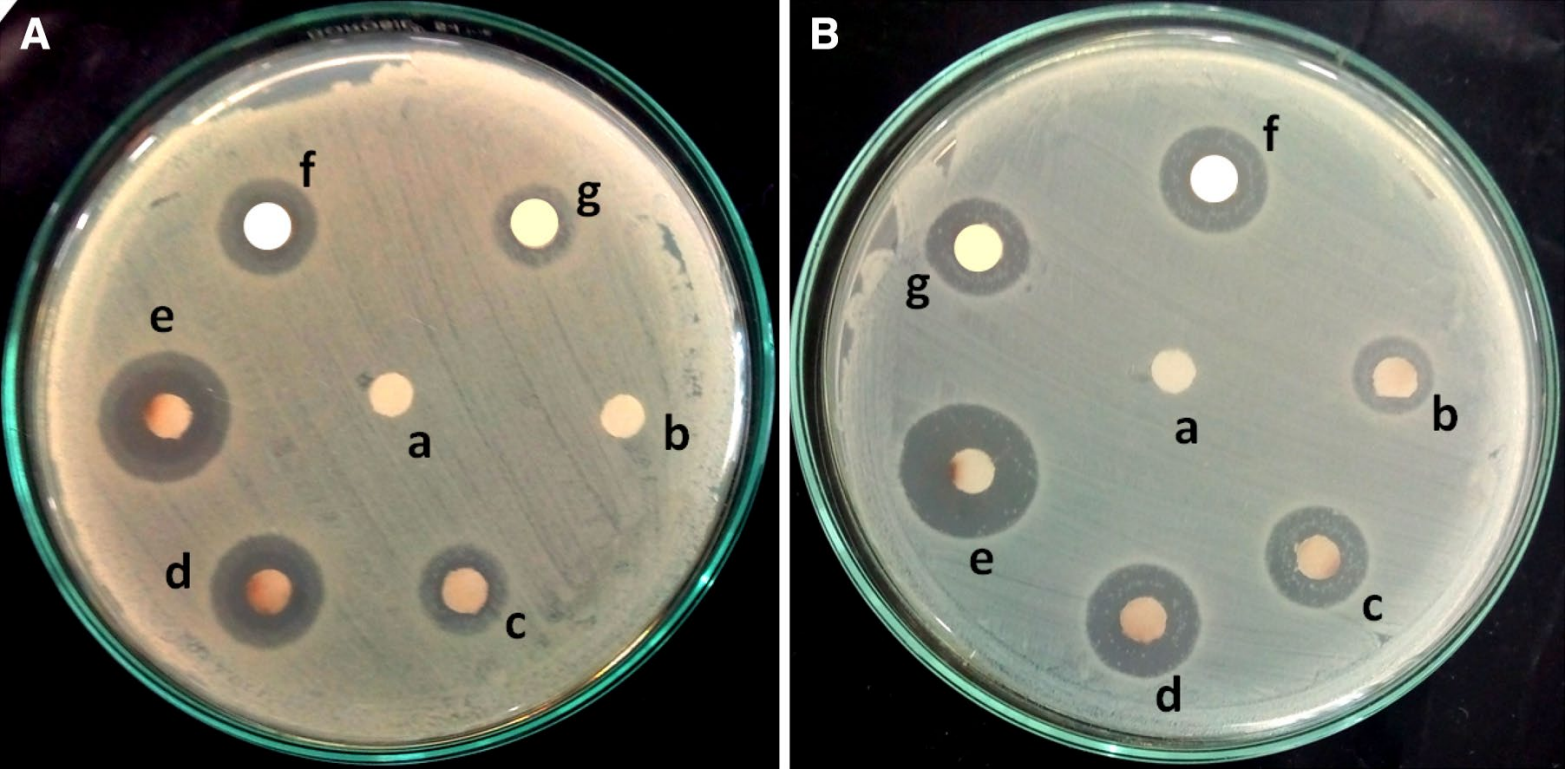
Zone of inhibition at different concentration of AgNPs against **A** *E. coli* and **B** *S. aureus*. *a* Cell free filtrate, *b* 25 ppm, *c* 50 ppm, *d* 75 ppm and *e* 100 ppm of AgNPs, *f* ampicillin (10 µg/disc) for *E. coli* and amikacin (30 µg/disc) for *S. aureus*, *g* 100 ppm of AgNO_3_

Table 1 shows MIC value of AgNPs against *E. coli* and *S. aureus*. MIC against both the bacterial strains was 100 ppm as demonstrated by broth dilution method. Furthermore, the antibacterial activity of AgNPs synthesized using CFF of *S*. *sclerotiorum* MTCC 8785 was evaluated against *E. coli* and *S. aureus* by bacterial growth kinetics study. The results depicted in Fig. 9 demonstrated the inhibition in the growth kinetics of test bacteria as compared to the cultures grown in absence of AgNPs. Reduction in the growth profile of *E. coli* (Fig. 9a) and *S. aureus* (Fig. 9b) was observed at 100 ppm of AgNPs as compared to the control sample. No growth was observed at 125 ppm of AgNPs. Furthermore, antibacterial activity was assessed by disc diffusion assay and zone of inhibition was depicted in Fig. 10. AgNPs showed antibacterial activity and inhibitory zone of 20 mm was recorded against *E. coli* at 100 ppm of AgNPs **(**Fig. 10Ae**)**. For *S. c* zone of inhibition at 100 ppm (Fig. 10Be) (Table 1). It may be due to perforation and lysis of AgNPs to the bacterial cell wall followed by generation of free radicals (Prabhu and Poulose 2012) and degradation of DNA (Duran et al. 2015). Zone of inhibition of *E. coli* and *S. aureus* againt AgNO_3_ and increasing concentration of AgNPs has been shown in Table 2. AgNO_3_ shown less zone of inhibition as compared to AgNPs at similar concentration suggested antibacterial activity is due to AgNPs. This can be due to reduction of AgNO_3_ into AgNPs which resulted in increased surface area that lead to better surface contact with bacteria and hence better bactericidal in nature (Prabhu and Poulose 2012). Ampicillin (10 µg/disc) and amikacin (30 µg/disc) were used as positive control against *E. coli* (Fig. 10Af) and *S. aureus* (Fig. 10Bf) respectively. Cell free filtrate was used as negative control (Fig. 10Aa, Ba). AgNPs showed maximum antibacterial activity as demonstrated by growth kinetics and disc diffusion assay at 100 ppm which is in agreement with MIC value.

**Table 1:** Minimum Inhibitory Concentration (MIC) of AgNPs against *E. coli* and *S. aureus*

S. no.	Bacterial strains	MIC of AgNPs (ppm)
1.	*E. coli*	100
2.	*S. aureus*	100

**Table 2:** Zone of inhibition of AgNPs, AgNO_3_ and standard antibiotics against *E. coli* and *S. aureus*

S. no.	Bacterial strains	Zone of inhibition (mm) ± SEM						
		AgNPs				AgNO_3_	Ampicillin (10 µg/disc)	Amikacin (30 µg/disc)
		25 ppm	50 ppm	75 ppm	100 ppm	100 ppm		
1.	*E. coli*	0	7 ± 0.4	15 ± 1.2	20 ± 1.3	11 ± 1.1	15 ± 0.9	–
2.	*S. aureus*	5 ± 0.3	9 ± 0.6	14 ± 1.6	19 ± 1.2	12 ± 0.8	–	17 ± 1.3

## Conclusion

 In conclusion, AgNPs can be synthesized using CFF of *S*. *sclerotiorum* MTCC 8785. Optimization of physical and cultural conditions revealed enhanced AgNPs synthesis in PDB grown fungi with 10 gm of biomass treated with 2 mM AgNO_3_ pH 11 and incubated at 80 °C. The green synthesized AgNPs were of 10 nm size and having protein as stabilizing agent under optimized conditions. AgNPs showed antibacterial activity against *E. coli* and *S. aureus*. Hence, green synthesis of AgNPs using *S*. *sclerotiorum* MTCC 8785 with potent antibacterial activities can be exploited on a large scale for medical application.

## Additional file

10.1186/s40064-016-2558-x Comparison of UV-Vis spectra of AgNPs before (A) and after purification (B).
