# Supplementary figures and images for: Process optimization for green synthesis of silver nanoparticles by Sclerotinia sclerotiorum MTCC 8785 and evaluation of its antibacterial properties
Source: Springerplus. 2016 Jun 24;5(1):861. doi: 10.1186/s40064-016-2558-x (PMC4920743; doi:10.1186/s40064-016-2558-x)

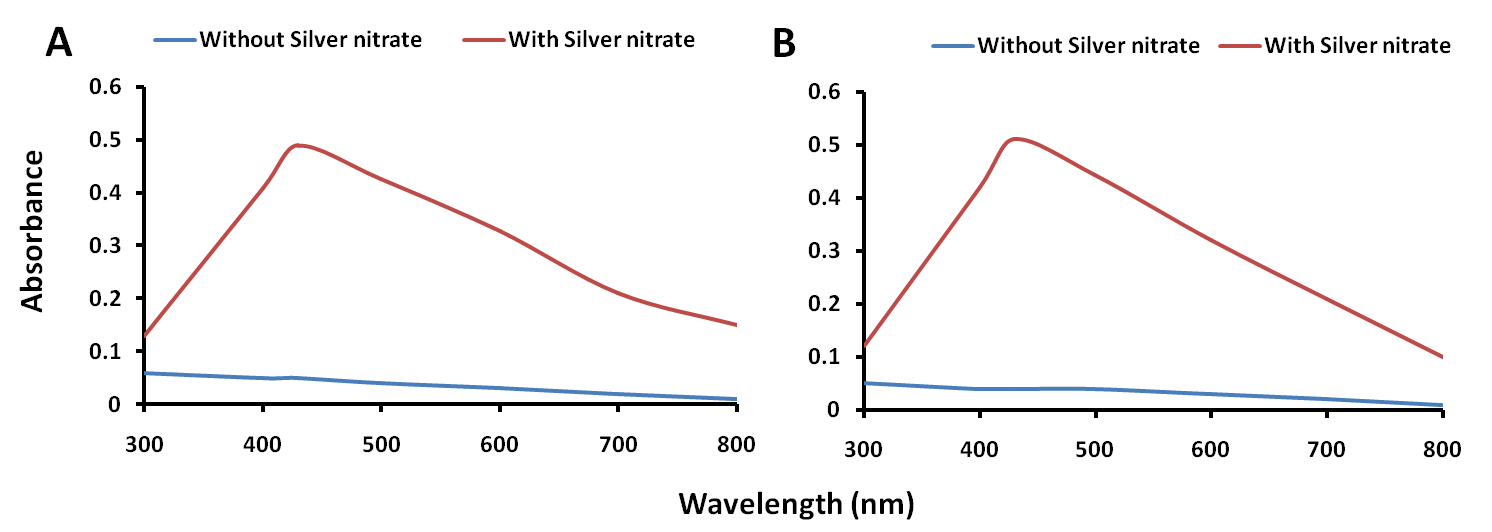


**Fig. S1 -** Comparison of UV-Vis spectra of AgNPs before (A) and after purification (B)

Supplement: Supplementary file 1 — 10.1186/s40064-016-2558-x Comparison of UV-Vis spectra of AgNPs before (A) and after purification (B). [file 40064_2016_2558_MOESM1_ESM.docx]
